# Indirect and direct routes to *C*-glycosylated flavones in *Saccharomyces cerevisiae*

**DOI:** 10.1186/s12934-018-0952-5

**Published:** 2018-07-09

**Authors:** Katherina Garcia Vanegas, Arésu Bondrup Larsen, Michael Eichenberger, David Fischer, Uffe Hasbro Mortensen, Michael Naesby

**Affiliations:** 1 Department of Biotechnology and Biomedicine, Technical University of Denmark, Søltofts Plads, Building 223, 2800 Kgs Lyngby, Copenhagen, Denmark; 2 Evolva SA, Duggingerstrasse 23, 4153 Reinach, Switzerland

**Keywords:** Vitexin, Isovitexin, Orientin, Isoorientin, Glycosyl *C*-transferase, Flavanone 2-hydroxylase

## Abstract

**Background:**

*C*-glycosylated flavones have recently attracted increased attention due to their possible benefits in human health. These biologically active compounds are part of the human diet, and the *C*-linkage makes them more resistant to hydrolysis and degradation than *O*-glycosides. In contrast to *O*-glycosyltransferases, few *C*-glycosyltransferases (CGTs) have so far been characterized. Two different biosynthetic routes for *C*-glycosylated flavones have been identified in plants. Depending on the type of *C*-glycosyltransferase, flavones can be glycosylated either directly or indirectly via *C*-glycosylation of a 2-hydroxyflavanone intermediate formed by a flavanone 2-hydroxylase (F2H).

**Results:**

In this study, we reconstructed the pathways in the yeast *Saccharomyces cerevisiae*, to produce some relevant CGT substrates, either the flavanones naringenin and eriodictyol or the flavones apigenin and luteolin. We then demonstrated two-step indirect glycosylation using combinations of F2H and CGT, to convert 2-hydroxyflavanone intermediates into the 6*C*-glucoside flavones isovitexin and isoorientin, and the 8*C*-glucoside flavones vitexin and orientin. Furthermore, we established direct glycosylation of flavones using the recently identified GtUF6CGT1 from *Gentiana triflora*. The ratio between 6*C* and 8*C* glycosylation depended on the CGT used. The indirect route resulted in mixtures, similar to what has been reported for in vitro experiments. In this case, hydroxylation at the flavonoid 3′-position shifted the ratio towards the 8*C*-glucosylated orientin. The direct flavone glycosylation by GtUF6CGT1, on the other hand, resulted exclusively in 6*C*-glucosides.

**Conclusions:**

The current study features yeast as a promising host for production of flavone *C*-glycosides, and it provides a set of tools and strains for identifying and studying CGTs and their mechanisms of *C*-glycosylation.

**Electronic supplementary material:**

The online version of this article (10.1186/s12934-018-0952-5) contains supplementary material, which is available to authorized users.

## Background

**Fig. 1:**
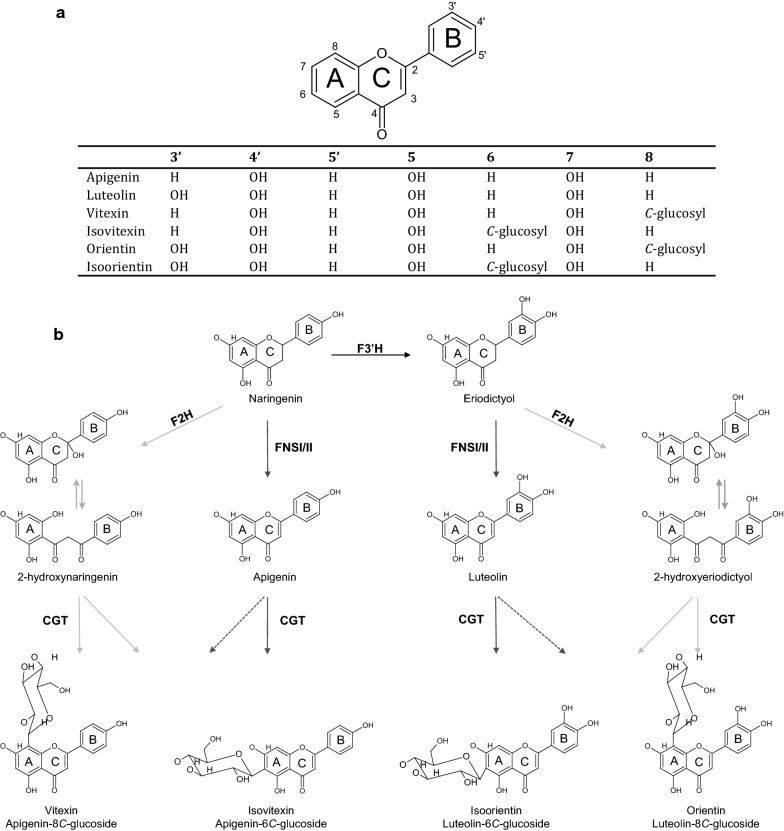
**a** Structures of some important flavones. **b** Predicted biosynthesis for *C*-glycosylated flavones from the common naringenin precursor. FNSI/II, flavone synthase 1 or 2; F2H, flavanone-2-hydroxylase; F3′H, flavanone-3′-hydroxylase CGT, *C*-glycosyltransferase. Broken line arrows represent hypothetical steps not demonstrated in this study. Equilibrium arrows indicate 2-hydroxylflavanones equilibrium with its open-circular form. Light grey arrows indicate the indirect *C*-glycosylation pathway and dark grey arrows shows the direct *C*-glycosylation pathway

Flavones constitute a subclass of flavonoids, found in fruits and vegetables [1], which has been associated with a range of human health-related benefits [2]. The basic flavone scaffold comprises a three ring-skeleton (Fig. 1a) with three functional groups: a C4 ketone, a conjugated C2–C3 double bond and, depending on the flavone, various numbers of hydroxyl groups [2, 3]. In plants the flavonoid scaffold is synthesized by condensation of two precursors derived from two different pathways of the primary metabolism, *p*-coumaroyl-CoA from the phenylalanine pathway and malonyl-CoA, an intermediate of fatty acid biosynthesis, to yield the common flavanone precursor naringenin [2, 4] (Additional file 1: Figure S1). Flavones are normally derived from the flavanones by the action of flavone synthase type I (FNS I), a 2-oxoglutarate dependent dioxygenase [5], or type II (FNS II) [6], a cytochrome P450 oxidase (CYP450) which introduce a C2–C3 double bond in the heterocyclic C-ring (Fig. 1b).

Various modifications of the flavone backbone result in a high degree of chemical diversity, resulting in different biological activities [2, 7]. One of the most common modifications is glycosylation, which can improve the biological activity and the solubility of the flavone [8, 9]. There are two main types of glycosylation, *O*-glycosylation and *C*-glycosylation, and the linkage of the glycosyl moiety to the flavone scaffold determines which type it is [10]. In *C*-glycosylation the linkage occurs directly between the glycosyl moiety and one of the carbon atoms of the flavone backbone [2]. *C*-glycosylation results in very stable molecules because the C–C bond linkage, unlike the O–C bond, is very resistant to acid hydrolysis and enzymatic glycosidase action [11, 12]. This has spurred an increased interest in *C*-glycosides for human health applications, including those related to metabolic syndrome [13, 14], since these molecules are expected to be more resistant to degradation in the human gastro-intestinal system, and therefore more orally bioavailable. In addition, these compounds are being investigated for prevention of certain cancers [15, 16].

*C*-glycosylated flavones are widespread in nature, and natural sources include cereals like rice, wheat, and maize where these glucosides are among the most abundant flavonoids [11, 17, 18]. Additional sources of a variety of flavone *C*-glycosides are for example bamboo [19], buckwheat [20], and flax [21]. The most commonly found *C*-glycosides are the mono-glucosides vitexin, isovitexin, orientin, and isoorientin derived from the common precursor naringenin (Fig. 1b).

The biosynthesis of flavone *C*-glycosides was studied in cereals, and somewhat surprisingly it was found that flavones themselves are not the direct substrate of *C*-glycosylation [11, 20, 22]. Instead, the substrate was shown to be the 2-hydroxyflavanone intermediate formed by a class of FNS II related flavanone 2-hydroxylases, belonging to the CYP93 family of enzymes [23]. Glycosylation is proposed to happen on an open form of the 2-hydroxyflavanone and a dehydratase has been implied to catalyse the leaving of the 2-hydroxy group [11, 20, 23].

Other natural sources of flavone *C*-glucosides are dicots like the gentians [24] and passion fruit [25], which contain high amounts of isoorientin. Very recently, Sasaki and co-workers identified a *C*-glycosyltransferase from *Gentiana triflora* which catalyses the direct *C*-glycosylation of flavones, including apigenin and luteolin [26]. No other enzyme has so far been reported to do this reaction.

Despite the potential human benefits of flavone *C*-glucosides, there are currently few reports of industrial scale production of these molecules from natural sources. This probably stems from the classical challenge of plant raw materials containing the desired compounds in low amounts and as part of complex mixtures. In turn, that would make production in an engineered, fermentable host an attractive alternative but there are so far no reports of de novo production of flavone *C*-glycosides in bacteria or yeast. Brazier-Hicks and Edwards co-expressed the OsF2H (CYP93G2) and the OsCGT from rice (*Oryza sativa*) in yeast, and by feeding naringenin to the culture they showed production of 8.2 mg/L of the 2-hydroxynaringenin glucoside, which was chemically converted to the corresponding flavone *C*-glycosides [27]. In contrast, production in yeast of naringenin from glucose has previously been reported [4, 28, 29] and the current study reports the reconstruction of full-length pathways to the four basic *C*-glucosides isovitexin, vitexin, isoorientin and orientin.

## Methods

### Chemicals

Chemical standards for detection and quantification of phloretic acid*, p*-coumaric acid, naringenin, luteolin, apigenin, vitexin, isovitexin, orientin, isoorientin and eriodictyol, were purchased from Sigma-Aldrich (St. Louis, Missouri, USA). We acquired standards for all the expected compounds (see Fig. 1 for details), except for the two 2-hydroxyflavanones, 2-hydroxynaringenin and 2-hydroxyeriodictyol. Neither of the two 2-hydroxy compounds was available for purchase from reliable suppliers and were anyway expected to be unstable due to spontaneous conversion into flavones by dehydration [22, 30].

### Strains and culture conditions

*Escherichia coli* (*E. coli*) XL10 Gold (Agilent, Santa Clara, California, USA) competent cells were used for subcloning of genes. After transformation *E. coli* cells were cultured at 37 °C for 12 h on Luria Broth (LB) plates prepared with 25 g/L of LB Broth with agar (Miller) and supplemented with 100 μg/mL ampicillin. Plasmid rescue cultivations were prepared using liquid LB media prepared with 25 g/L LB Broth (Miller) and supplemented with 100 μg/mL ampicillin.

Yeast strains used in this study were all direct descendants of *S. cerevisiae* S288C strain NCYC 3608 (NCYC, Norwich, United Kingdom). One descendant, the BG strain described earlier [31], was the basis of strains used in this study (Table 1). The BG strain was modified to replace the non-functional gal2 gene with a functional allele from *S. cerevisiae* SK1 strain NCYC 3615 (NCYC). Further, the *ARO3* gene was deleted and replaced by mutant *ARO4* and *ARO7* genes, encoding de-regulated versions of these enzymes [32] resulting in strain EYS4988. To prevent degradation of precursors of the heterologous flavonoid pathway, the host genes *ARO10*, *PAD1*, and *FDC1* were all deleted to create strain EVST27089 (Additional file 1: Figure S2). Finally, the entire naringenin pathway was integrated into the XI-3 site [33] by in vivo homologous recombination essentially as described by Shao et al. [34] to create strain EVST28856. This strain was used to express the flavone glucoside pathways on Homologous Recombination Technology (HRT) plasmids [31] (see plasmid assembly below). A list of genes used in this study is provided in Additional file 1: Table S1.

**Table 1:** Main yeast strains used in this work

Strains	Genotype	Source
BG	*MATα his3Δ0 leu2Δ0 ura3Δ0 hoΔ0 gal2Δ0::GAL2 cat5Δ0::CAT5(J91* *M) mip1Δ0::MIP1(A661T) sal1Δ0::SAL1(G403L)*	[31]
EYS4988	*MATα his3Δ0 leu2Δ0 ura3Δ0 hoΔ0 gal2Δ0::GAL2 cat5Δ0::CAT5(J91* *M) mip1Δ0::MIP1(A661T) sal1Δ0::SAL1(G403L) aro3Δ::LoxP/pTEF1*-*ARO4(K229L)*-*tCYC1/pPGK1*-*ARO7(T266L)*-*tADH1*	This study
EVST27089	*MATα his3Δ0 leu2Δ0 ura3Δ0 hoΔ0 gal2Δ0::GAL2 cat5Δ0::CAT5(J91* *M) mip1Δ0::MIP1(A661T) sal1Δ0::SAL1(G403L) aro3Δ::LoxP/pTEF1*-*ARO4(K229L)*-*tCYC1/pPGK1*-*ARO7(T266L)*-*tADH1 aro10Δ::LoxP pad1Δ/fdc1Δ::LoxP*	This study
EVST28856	*MATα his3Δ0 leu2Δ0 ura3Δ0 hoΔ0 gal2Δ0::GAL2 cat5Δ0::CAT5(J91M) mip1Δ0::MIP1(A661T) sal1Δ0::SAL1(G403L) aro3Δ::LoxP/pTEF1*-*ARO4(K229L)*-*tCYC1/pPGK1*-*ARO7(T266L)*-*tADH1 aro10Δ::LoxP pad1Δ/fdc1Δ::LoxP XI*-*3::loxP/pGPD1*-*MdCHSc_co*-*tCYC1/pPGK1*-*MsCHI_co*-*tADH2/pTEF1*-*At4CL2_co*-*tENO2/pPDC1*-*AtPAL2_co*-*tFBA1/pTEF2*-*AmC4H_co*-*tPGI1/pPYK1*-*ScCPR*-*tADH1*	This study

Yeast cultures were grown in Synthetic Complete (SC) Drop Out medium (Formedium, Hunstanton, United Kingdom) prepared with 47 g/L SC,-His,-Leu,-Ura Drop Out powder. Depending on auxotrophic selection markers, the SC medium was supplemented with histidine (76 mg/L), leucine (380 mg/L), and/or uracil (76 mg/L). The pH was adjusted to 5.8 with hydrochloric acid, and the medium was then supplemented with 6.7 g/L yeast nitrogen base without amino acids (ThermoFischer, Waltham, MA, USA) and 20 g/L d-(+)-glucose. For preparing plates, 20 g/L of agar was added. Cultures were grown in half-deep 96-well plates in a Kühner ISF-1-W shaker (Kühner, Birsfelden, Switzerland) at 30 °C, 300 RPM and 50 mm amplitude. Cultures were prepared by diluting a pre-culture to an optical density at 600 nm (OD600) of 0.1, and grown, in a final volume of 300 µL, at 30 °C, 300 RPM, and 50 mm amplitude for 72 h before being extracted and analysed.

### Assembly of plasmids by in vivo homologous recombination

Sequences of selected genes (Additional file 1: Table S1) were codon optimized for expression in *S. cerevisiae* and synthesized by GeneArt (ThermoFisher). During synthesis, all genes were provided with a *Hin*dIII restriction site and an AAA Kozak sequence at the 5′-end and a *Sac*II restriction site at the 3′-end. These sites were used for cloning into yeast expression cassettes of HRT vectors pEVE2176, pEVE2177 or pEVE2178 for multigene plasmid assembly by in vivo homologous recombination as described earlier [31]. Additional file 1: Figure S3 gives a schematic representation of the HRT technology and Additional file 1: Table S2 provides a detailed description of all plasmids used in this study. Briefly, the HRT vectors contained 60 bp recombination tags flanking the expression cassettes (promoter and terminator) into which the genes, or in some cases non-coding stuffer fragments, were cloned. The tagged cassettes were nested between a set of *Asc*I restriction sites, used to release them from the vector backbone. Plasmids containing the expression cassettes, as well as helper fragments containing all elements required for single copy plasmid replication (pEVE1968) and selection (pEVE4730 or pEVE4729), and a closing linker (pEVE1973 or pEVE1916), were combined and digested with *Asc*I in a single 10 μL reaction and used directly to transform yeast in a standard Li Acetate transformation [35]. Subsequently, the overlapping HRT recombination tags, flanking each fragment, directed the in vivo plasmid self-assembly.

### Sample preparation and analytical method

After 72 h of cultivation, 150 μL of culture broth was transferred into a deep 96-well plate and diluted with 1 volume 100% methanol. The diluted broth was incubated for 10 min at 30 °C, 300 RPM, 50 mm amplitude and clarified by centrifugation for 5 min at 4000*g*. The cleared lysate was diluted 20 times in 50% methanol in water, transferred into FrameStar^®^ 96 Well Skirted Plates (4titude^®^, Surrey, United Kingdom), and analysed by UPLC–MS on a Waters Acquity system coupled to a Waters Xevo G2 XS Tof mass detector (Milford, MA, USA). The system was equipped with a Waters Acquity UPLC^®^ BEH C18 column (1.7 µm, 2.1 mm × 50 mm) and the column temperature was kept at 55 °C. The gradient was composed of the two phases, water (A) and acetonitrile (B), both buffered with 0.1% formic acid. Initially, the B phase was kept constant at 10% for 0.1 min, and then the B fraction was increased from 10 to 25% over 2.4 min, and from 25 to 100% in 0.5 min. Finally, we performed a column wash procedure for 0.5 min with 100% phase B followed by equilibration in 10% B for 0.5 min. The flow rate was kept at 0.8 mL/min during the complete gradient program.

The mass analyser was set to operate in negative ion mode. The nebulization gas flow was set to 1000 L/h at a temperature of 500 °C. The cone gas flow was set to 100 L/h and the source temperature was set to 150 °C. The capillary voltage and cone voltage were set to 1000 and 40 V, respectively. For each compound of interest we calculated peak areas on the extracted ion chromatograms of the respective [M–H]^−^ ions using a mass window of 0.02 Da. Compounds were quantified using a linear calibration curve with authentic standards ranging from 0.03 to 4 mg/L for all compounds.

## Results

### Construction of yeast strains for flavanone and flavone production

In order to test *C*-glycosylation of both flavanones and flavones we created the four strains NAR1, ERI1, API1, and LUT1 producing the flavanones naringenin and eriodictyol, and the corresponding flavones apigenin and luteolin, respectively. The NAR1 strain was based on the EVST28856 strain, which already had the naringenin pathway, comprising the genes *AtPAL2*, *AmC4H*, *ScCPR1*, *At4C2L*, *MsCHI*, and *MdCHS,* integrated (Additional file 1: Figure S1). This strain had been further optimized for aromatic amino acid production, by incorporating feedback insensitive versions of the native *ARO4* and *ARO7* genes [32]. To prevent degradation of pathway precursors, the host genes *ARO10*, *PAD1*, and *FDC1* had been deleted [4, 36] (Additional file 1: Figure S2).

The NAR1 strain was created by in vivo assembly of a low-copy HRT vector comprising the *AtCPR1* gene. Specifically, the cassettes for construction of the HRT plasmid were assembled in the following order (see Additional file 1: Figure S3 and Table S2): a *URA3* marker cassette for selection (from pEVE4730), an ARS4/CEN6 cassette (from pEVE1968), two non-coding “stuffer sequence” cassettes (from pEVE2176 to pEVE27453), the *AtCPR1* gene (from pEVE4012) cassette, and finally the EZ closing linker (from pEVE1916), which is used to close the plasmid as it fuses to pEVE4012 and pEVE4730 (Additional file 1: Figure S3). The HRT plasmids in the ERI1, API1, and LUT1 strains were constructed in the same manner as NAR1 except that the relevant genes *AmFNSII* (pEVE23312) [37] and *PhF3′H* (pEVE3999) [38] substituted the empty cassettes. Hence, for the API1 strain (containing *AtCPR1* and *AmFNSII*) pEVE2176 was replaced by pEVE23312. For the ERI1 strain (containing *AtCPR1* and *PhF3′H*) pEVE27453 was replaced by pEVE3999. For the LUT1 strain (containing *AtCPR1*, *AmFNSII*, and *PhF3′H*), pEVE2176 and pEVE27453 were replaced by pEVE23312 and pEVE3999, respectively. The four strains were cultured for 72 h and analysed for production of the expected flavonoids.

**Fig. 2:**
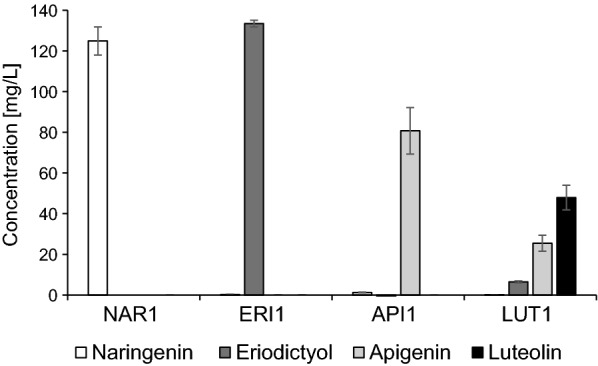
Basic strains for testing both indirect and direct *C*-glycosylation pathways. Production of naringenin (white), eriodictyol (dark grey), apigenin (light grey), and luteolin (black) by strains NAR1, ERI1, API1 and LUT1, respectively. Represented are averages and standard deviations of four independent cultures

The NAR1 strain produced 124.91 ± 6.94 mg/L naringenin, the ERI1 strain produced 133.43 ± 1.63 mg/L eriodictyol, and the API1 strain produced 80.74 ± 11.38 mg/L apigenin (Fig. 2). The LUT1 strain produced 47.90 ± 6.05 mg/L luteolin, as well as around 25 mg/L of apigenin (Fig. 2). All four strains accumulated residual *p*-coumaric acid, between 18 and 19 mg/L in the NAR1 and ERI1 strains, and 12–13 mg/L in API1 and LUT1 strains, but no other direct intermediates were detected.

The four basic strains developed in this section, thus, set the stage for testing *C*-glycosylation of flavones, strains NAR1 and ERI1 by the indirect route and strains API1 and LUT1 by the direct route.

### Indirect *C*-glycosylation of flavones via 2-hydroxyflavanones

In some monocots such as the cereals rice, sorghum, and maize, *C*-glycosylated flavones are the major class of flavonoids [11], and recent work has shown that these flavones are derived from 2-hydroxylated flavanones. Furthermore, in these plants, CYP450 enzymes have been shown to hydroxylate flavanones at the 2-position, and the 2-hydroxyflavanones then serve as substrate for the *C*-glycosyltransferases. Three flavanone 2-hydroxylases including the rice (*Oryza sativa*) OsCYP93G2 [23], the sorghum (*Sorghum bicolor*) SbCYP93G3 [39], and the maize (*Zea mays*) ZmCYP93G5 [40] were selected for providing 2-hydroxylated flavanones for *C*-glycosylation.

Although CGTs have been identified from the monocots rice and maize, the latter of these has been reported to also show *O*-glycosylating activity on 2-hydroxylated flavanones [12]. However, also dicots are known to produce flavone *C*-glycosides, both of the 6*C* and 8*C* types, and it seems that plants acquired the ability to *C*-glycosylate 2-hydroxyflavanones before the monocot–dicot species split [22]. Hence, CGTs would be expected to accept 2-hydroxyflavanones independent on which F2H, dicot or monocot, provides them. Thus, two dicot CGTs, the UGT708C2 [20] from buckwheat (*Fagopyrum esculentum*) and the UGT708D1 [22] from soybean (*Glycine max*), with no reported *O*-glycosylation activity, were chosen for producing the four corresponding *C*-glycosides from either 2-hydroxynaringenin or 2-hydroxyeriodictyol.

**Fig. 3:**
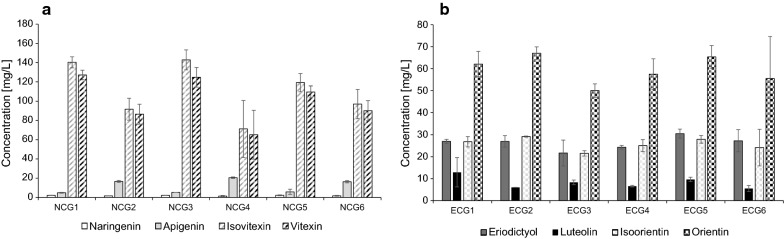
Indirect *C*-glycosylation. **a** NCG strains production of naringenin (white), apigenin (light grey), isovitexin (grey hatching) and vitexin (black hatching). **b** ECG strains production of eriodictyol (dark grey), luteolin (black), isoorientin (grey checker) and orientin (black checker). Represented are averages and standard deviations of four independent cultures

By in vivo assembly of a second HRT plasmid, combinations of F2H and CGTs were introduced into strain NAR1 to create a set of strains NCG1–NCG6, and into strain ERI1 to create a set of strains ECG1–ECG6 (see Table 2). For the second HRT plasmid a different backbone was used (pEVE4729) to allow co-selection with the first plasmid, using a different selectable marker (see Additional file 1: Table S2). Further, it comprised one empty cassette (pEVE2178). These 12 new strains were cultured and analysed for production of flavone *C*-glucosides (Fig. 3).

**Table 2:** List of the two strain sets for indirect production of *C*-glycosylated flavones

NCG strain set	ECG strain set	Gene combinations on corresponding HRT plasmid
NCG1	ECG1	*OsCYP93G2* and *UGT708C2*
NCG2	ECG2	*OsCYP93G2* and *UGT708D1*
NCG3	ECG3	*SbCYP93G3* and *UGT708C2*
NCG4	ECG4	*SbCYP93G3* and *UGT708D1*
NCG5	ECG5	*ZmCYP93G5* and *UGT708C2*
NCG6	ECG6	*ZmCYP93G5* and *UGT708D1*

NCG strains were analysed for naringenin and the corresponding *C*-glucosides isovitexin (apigenin-6*C*-glucoside) and vitexin (apigenin-8*C*-glucoside) (Fig. 3a and Additional file 1: Figure S4). As we expected the 2-hydroxynaringenin intermediate to be potentially unstable, the strains were also analysed for the aglycone apigenin. All strains accumulated very little naringenin, less than 2.3 mg/L, and relatively small amounts of apigenin, less than 20.5 mg/L. In contrast, they all produced large quantities of both isovitexin and vitexin. Although not statistically significant, the trend was towards slightly more of the 6*C* glucoside isovitexin. With all three F2H enzymes the UGT708C2 gave the highest titres, reaching around 140 mg/L isovitexin and around 125 mg/L of vitexin in combination with either OsCYP93G2 (strain NCG1) or SbCYP93G3 (strain NCG3).

ECG strains were similarly analysed for eriodictyol and the corresponding *C*-glucosides isoorientin (luteolin-6*C*-glucoside) and orientin (luteolin-8*C*-glucoside), as well as other potential intermediates of this pathway. As seen in Fig. 3b, these strains accumulated relatively high amounts of non-reacted eriodictyol, up to around 30 mg/L, and some non-glycosylated luteolin, up to about 13 mg/L. Surprisingly, all ECG strains exhibited a clear preference for producing the 8*C*-glycosylated orientin versus the 6*C*-glycosylated isoorientin. In strain ECG2, for example, a level of 67.03 ± 2.92 mg/L orientin was reached, compared to 29.19 ± 0.24 mg/L of isoorientin.

The ECG strains also accumulated products derived from naringenin via 2-hydroxynaringenin, i.e. more than approx. 50 mg/L isovitexin and 48 mg/L vitexin (Additional file 1: Table S3). This reflects the competition between two branches of the biosynthetic pathway, in which F2H and F3′H compete for the common substrate naringenin, leading to formation of either 2-hydroxynaringenin or, via eriodictyol, to 2-hydroxyeriodictyol, with both 2-hydroxylated compounds being substrates for the CGTs (see Fig. 1).

In all the NCG and ECG strains additional peaks were detected (Additional file 1: Table S4). Interestingly, the m/z value of two of these peaks corresponded to the calculated mass of 2-hydroxyflavanone-*C*-glucosides and these peaks most likely represent the hypothesized intermediates for the *C*-glycosylated flavones. This notion is supported by the fact that NCG strains gave rise only to compounds predicted to derive from naringenin, whereas in the ECG strains peaks expected to derive from both naringenin and eriodictyol were detected. Unfortunately, these compounds could not be quantified due to lack of authentic reference compounds.

### Direct *C*-glycosylation of flavones by *Gentiana triflora* CGT

**Fig. 4:**
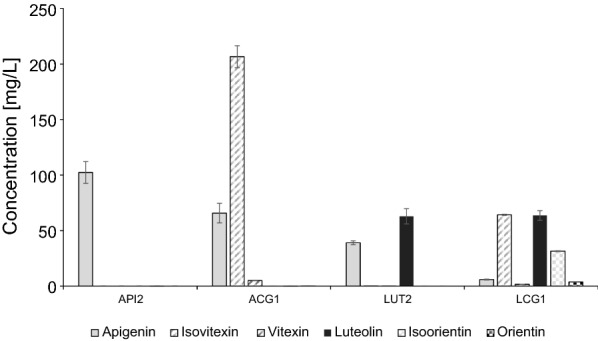
Direct *C*-glycosylation. Production of apigenin (light grey), isovitexin (grey hatching), vitexin (black hatching), luteolin (black), isoorientin (grey checker) and orientin (black checker) by strains API2 (no CGT), ACG1 (with GtUF6CGT1), LUT2 (no CGT) and LCG1 (with GtUF6CGT1). Represented are averages and standard deviations of four independent cultures

Recently a CGT from *G. triflora* was characterized and shown to *C*-glycosylate flavones directly at the 6*C*-position of both apigenin and luteolin [26]. This CGT, known as GtUF6CGT1, was cloned and expressed on a second HRT plasmid in both the API1 and LUT1 strains. As above, the backbone used for the HRT plasmid (pEVE4729) was different from that of the first plasmid, and this time also a different closing linker was used (pEVE1973). The strains were named ACG1 and LCG1, respectively. An empty cassette (pEVE2176) was used instead of the CGT, to create the control strains API2 and LUT2. These four new strains were analysed for production of flavones and their corresponding *C*-glycosides (Fig. 4, Additional file 1: Table S5). When GtUF6CGT1 was expressed in the ACG1 strain, we recorded production of more than 200 mg/L (206.47 ± 2.92 mg/L) of the apigenin-6*C*-glucoside isovitexin, and almost none of the 8*C*-glucoside vitexin. In the LCG1 strain we observed a similar preference for the 6*C*-position, and detected both isovitexin (apigenin-6*C*-glucoside) and isoorientin (luteolin-6*C*-glucoside) of more than 60 mg/L (64.24 ± 0.51 mg/L) and 30 mg/L (31.47 ± 0.38 mg/L), respectively. Less than 4 mg/L of orientin was detected and essentially no vitexin. More than 63 mg/L of luteolin, and close to 6 mg/l apigenin accumulated in the culture broth (Additional file1: Table S5). These high levels of apigenin and isovitexin obviously reflects an insufficient F3′H activity in the biosynthetic pathway, something that was evident already in the parent LUT1 strain, but which seems to be exacerbated by the addition of the CGT. The fact that almost all apigenin gets glycosylated, whereas more than two-thirds of luteolin remains non-glycosylated, would indicate a preference by the glycosyltransferase for apigenin over luteolin, thereby creating an extra pull towards isovitexin. This is in contrast to what has been reported in vitro, where the partially purified enzyme showed higher activity on luteolin compared to apigenin [26]. Sasaki and co-workers reported that GtUF6CGT1 accepts only apigenin and luteolin as substrates, but not other flavonoids. In agreement, we found that NAR1 and ERI1 strains expressing the GtUF6CGT1, in the presence or absence of F2H, did not produce any detectable glycosylation products (data not shown).

## Discussion

### Production of flavanones and flavones

To provide the relevant substrates for production of flavone *C*-glycosides we constructed the four parental strains NAR1, ERI1, API1, and LUT1. The NAR1 strain produced around 125 mg/L naringenin, and in the ERI1 strain this was efficiently hydroxylated at the 3′-position by *PhF3′H*. The conversion of naringenin to apigenin by *AmFNSII* was less efficient, and only around two-thirds of naringenin was oxidized to apigenin in the API1 strain. To improve this situation perhaps a more active FNS could be identified, or the copy number of *AmFNSII* could be increased. However, the fact that hardly any naringenin was left in the culture might suggest other issues, e.g. production of unstable intermediates or derailment products. Similarly, the LUT1 strain produced less amount of flavone than expected and, again, this could be due to incomplete conversion by the FNS. Further, the relatively high residual level of apigenin would reflect a competition for naringenin between F3′H and FNS (see Fig. 1b), combined with the expected low or no affinity of F3′H for apigenin. In the flavonoid pathway it is generally assumed that F3′H acts only on flavanones and dihydroflavonols. It suggests that higher expression of F3′H might help shift the balance towards higher production of eriodictyol and, eventually, luteolin.

### Production of flavone *C*-glycosides

Several papers recently reported the biosynthesis of flavone *C*-glucosides in a two-step process, in which the actual substrate of CGTs is the open form of 2-hydroxyflavanones, the product of F2H [11, 20, 23]. In vitro, the glycosylated molecule would then ring-close, with concomitant loss of the OH-group, to form a mix of 6*C*- and 8*C*-glyccosides. The analysis of these products is challenging, since under many LC-MS conditions they will run as a single peak with the same mass. When eventually separated, they mostly show an approx. 1:1 ratio of the two glycosides [20, 22]. However, this ratio between 6*C* and 8*C* likely reflects only the in vitro situation, and it has been suggested that *in planta* the dehydration is an enzymatic process, involving a dehydratase, which directs the preferential formation of one isomer over the other. Brazier-Hicks and co-workers reported that incubating the rice OsCGT with the substrate 2,5,7-trihydroxyflavanone produced the corresponding 2,5,7-hydroxyflavanone-*C*-glucoside, which would spontaneously decompose to the 6*C* and 8*C* glucosides in the ratio of 0.5:1. In contrast, with a crude protein extract from rice cell cultures the formation of the 6*C*-glucoside would increase over time. They concluded that this was due to an enzymatic dehydratase activity [11]. Nagatomo and co-workers reported that incubating a recombinant UGT708C1 with 2-hydroxynaringenin resulted in a 6*C*:8*C* ratio of 1.37:1 in acid treated extracts, compared to a ratio of 1.87:1 in extracts from buckwheat cotyledons [20]. They concluded that this difference could only be explained by an enzymatic activity in the plant. Despite these results, a specific dehydratase enzyme has still to be identified and characterized.

Here, when F2Hs and CGTs were combined in the NAR1 strain we observed a good conversion into isovitexin and vitexin with a slight preference for isovitexin. These results are in line with in vitro results and, hence, indicates that there is no native dehydratase activity in yeast. Rather, the dehydration is likely spontaneous and driven by the acidification of the growth medium to which the 2-hydroxynaringenin-glucoside is readily secreted [27]. Additionally, small amounts of apigenin were detected, which could be due to residual FNS activity of F2H or spontaneous dehydration of the 2-hydroxygroup to form the C2–C3 double bond. We also detected 2-hydroxynaringenin and its glycoside 2-hydroxynaringenin-glucoside, but these compounds could not be quantified due to lack of standards (Additional file 1: Table S4). However, the detection of these compounds indicates some stability of the 2-OH group, in particular after glycosylation. Similar results were reported earlier, where yeast co-expressing F2H and CGT, and being fed with naringenin, produced predominantly 2-hydroxynaringenin glucoside, which accumulated in the growth medium [27]. However, as seen in vitro [11, 23], we would expect the eventual spontaneous dehydration of these compounds due to the gradual acidification of the medium, which after 72 h was around pH 3–4.

When the ERI1 strain was used for expressing combinations of F2Hs and CGTs the production of isovitexin and vitexin exhibited the same ratio between 6*C* and 8*C* glycosylation as seen in all the NCG strains (Additional file 1: Table S3). These glucosides were derived from residual naringenin, before this could be hydroxylated to eriodictyol. However, when eriodictyol was the direct substrate, the combined expression of F2H and CGT produced the 6*C*-glycosylated isoorientin and the 8*C*-glycosylated orientin in the ratio of approx. 1:2 as measured in the growth medium. Roughly the same ratio was seen for both of the two CGTs and, assuming the absence of enzymatic dehydratase activity, would suggest a propensity of the free, open conformation of 2-hydroxyeriodictyol to ring-close with the glucose attached at the 8*C*-position. We speculate that this conformation would be preferred due to some slightly stabilizing interactions, e.g. hydrogen bonding between the 3′-hydroxy group and the 6*C*-hydroxy group of the glucose, during ring closure. Also in all the ECG strains we detected compounds with the predicted mass of 2-hydroxyeriodictyol and its glucoside, as well as the corresponding derivatives of naringenin (Additional file 1: Table S4). As with the NCG strains, we detected residual amounts of flavones, specifically luteolin.

In contrast to the indirect 2-step *C*-glycosylation, expression of GtUF6CGT1 in strains ACG1 and LCG1 resulted almost exclusively in the 6*C*-glycosides of these flavones (Fig. 4). This is similar to what was reported by Sasaki and co-workers [26], and was therefore expected. We confirmed that in yeast, this enzyme retains its specificity for flavones, and does not accept flavanones. By further increasing the CGT activity in the ACG1 strain, we believe it should be possible to convert the remaining flavone to obtain an even higher amount of pure isovitexin. In addition, production of pure isoorientin should be achievable with this CGT, although some challenges remains. Firstly, the production of apigenin, and its *C*-glucosides, must be prevented by improving the flux towards eriodictyol. And secondly, the glycosylation of luteolin must be improved, e.g. by further overexpression of GtUF6CGT1 or by optimizing media and culture condition which may improve the specific activity of this enzyme. In any case, further progress would involve balancing the flux of the entire biosynthetic pathway.

## Conclusions

The yeast *S. cerevisiae* is an attractive production host for plant secondary metabolites due to its ability to efficiently express most plant genes, including CYP450s, its amenability to metabolic engineering, and the extensive industrial experience acquired regarding its fermentation and downstream processing. In the current study, we show that *S. cerevisiae* can also be used as an efficient platform for the identification of *C*-glycosyltransferases. Considering the industrial potential of *S. cerevisiae*, we believe that this yeast would make an excellent host for production of commercially relevant flavone *C*-glycosides.

Based on previous results and those presented here, it would seem relatively straightforward to establish isovitexin production by fermentation of yeast, using the 6*C*-specificity of GtUF6CGT1. More specific production of isoorientin could possibly be achieved by various engineering solutions, such as channelling the substrate via enzyme fusions, or by spatiotemporal separation of the required enzymatic steps. Alternatively, more specific enzymes could possibly be identified and/or engineered. The current strains provide an ideal set-up to allow screening for the desired activity.

Production of the 8*C*-glycosides looks more challenging. Perhaps a homolog of GtUF6CGT1, with the opposite specificity can be found. Unfortunately, no close homologs of the GtUF6CGT1 have so far been reported, nor are there any obvious candidates in public sequence databases. Alternatively, the two-step pathway would have to be improved. That, in turn, would likely involve further studies on the ring-closure mechanism, and/or identification of the elusive dehydratase. Also for such studies, the strains presented here would be useful, either as a screening tool or for more detailed studies of the *C*-glycosylation mechanisms.

Clearly, the current proof of concept study provides only the first step towards commercial production of these compounds, and huge efforts will have to go into both fermentation scale-up and downstream process development before this is a reality. However, we believe that researchers and engineers will eventually be able to fully control the biosynthetic pathway leading to specific flavone *C*-glycosides, allowing cost efficient production of any desired molecule. This will provide the pure compounds needed to study their potential health benefits, as well as afford the basis for potential commercial applications e.g. as nutritional supplements or functional ingredients in processed foods and beverages.

## Additional file

**Additional file 1: Figure S1.** De novo naringenin pathway in *S. cerevisiae* derived from phenylalanine and three malonyl-CoA. **Figure S2.** Host modifications in the naringenin strain. **Figure S3.** One-step in vivo assembly using Homologous Recombination Technology (HRT) in *S. cerevisiae*. **Figure S4.** Quantification of vitexin and isovitexin. **Table S1.** Synthetic and codon optimized genes, used in this study. **Table S2.** Main plasmids used in this study. **Table S3.** Indirect biosynthetic pathway. **Table S4.** Additional intermediates of the indirect biosynthetic pathway. **Table S5.** Direct biosynthetic pathway.
