# Correction to: Indirect and direct routes to *C*-glycosylated flavones in *Saccharomyces cerevisiae*

**DOI:** 10.1186/s12934-018-0967-y

**Published:** 2018-07-28

**Authors:** Katherina Garcia Vanegas, Arésu Bondrup Larsen, Michael Eichenberger, David Fischer, Uffe Hasbro Mortensen, Michael Naesby

**Affiliations:** 1 Department of Biotechnology and Biomedicine, Technical University of Denmark, Søltofts Plads, Building 223, 2800 Kgs Lyngby, Copenhagen, Denmark; 2 Evolva SA, Duggingerstrasse 23, 4153 Reinach, Switzerland

## Correction to: Microb Cell Fact (2018) 17:107 10.1186/s12934-018-0952-5

Upon publication of this article [1], it was brought to our attention that revised Fig. 1 supplied by the author during proof correction was unfortunately not presented in the original version of the article. The revised Fig. 1 is given in this erratum.

The original article has been corrected.

**Fig. 1:**
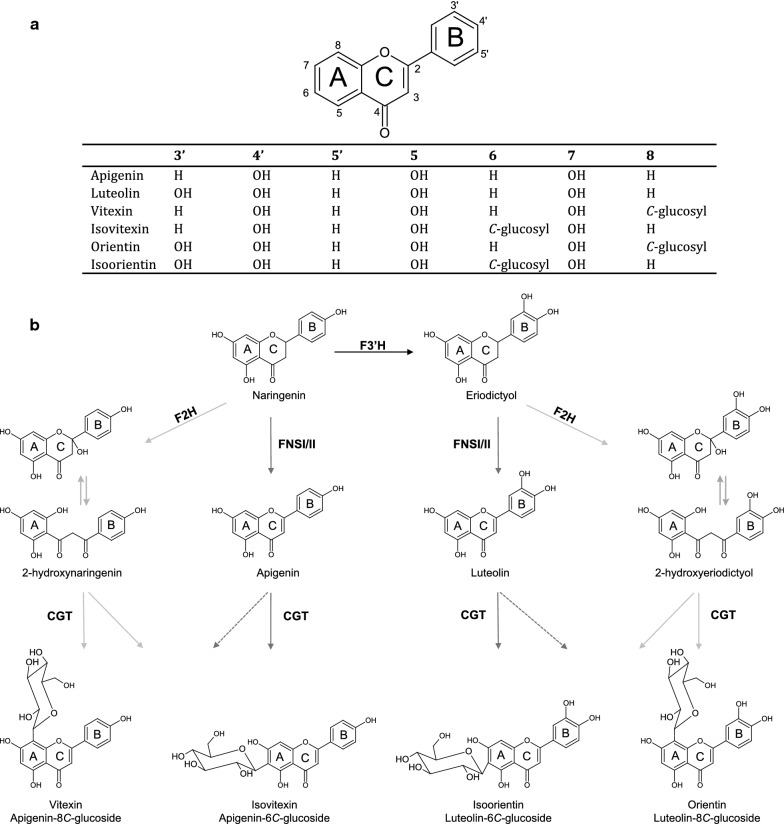
**a** Structures of some important flavones. **b** Predicted biosynthesis for *C*-glycosylated flavones from the common naringenin precursor. FNSI/II, flavone synthase 1 or 2; F2H, flavanone-2-hydroxylase; F3′H, flavanone-3′-hydroxylase CGT, *C*-glycosyltransferase. Broken line arrows represent hypothetical steps not demonstrated in this study. Equilibrium arrows indicate 2-hydroxylflavanones equilibrium with its open-circular form. Light grey arrows indicate the indirect *C*-glycosylation pathway and dark grey arrows shows the direct *C*-glycosylation pathway
