# Supplementary material for: Indirect and direct routes to C-glycosylated flavones in Saccharomyces cerevisiae
Source: Microb Cell Fact. 2018 Jul 9;17:107. doi: 10.1186/s12934-018-0952-5 (PMC6036675; doi:10.1186/s12934-018-0952-5)
Supplement: Supplementary file 1 — Additional file 1: Figure S1. De novo naringenin pathway in S. cerevisiae derived from phenylalanine and three malonyl-CoA. Figure S2. Host modifications in the naringenin strain. Figure S3. One-step in vivo assembly using Homologous Recombination Technology (HRT) in S. cerevisiae. Figure S4. Quantification of vitexin and isovitexin. Table S1. Synthetic and codon optimized genes, used in this study. Table S2. Main plasmids used in this study. Table S3. Indirect biosynthetic pathway. Table S4. Additional intermediates of the indirect biosynthetic pathway. Table S5. Direct biosynthetic pathway. [file 12934_2018_952_MOESM1_ESM.docx]

# Additional file 1: Indirect and direct routes to *C*-glycosylated flavones in *Saccharomyces cerevisiae*

**Author’s information**

Katherina Garcia Vanegas – KGV - kgava@bio.dtu.dk^1^

Arésu Bondrup Larsen – ABL – aresul@evolva.com^2^

Michael Eichenberger - ME - michaele@evolva.com^2^

David Fischer - DF - davidf@evolva.com^2^

Uffe Hasbro Mortensen – UHM - um@bio.dtu.dk^1^

Michael Naesby - MN - michaeln@evolva.com^2*^ - Corresponding author

^1^Department of Biotechnology and Biomedicine, Technical University of Denmark, Søltofts Plads, Building 223, 2800 Kgs. Lyngby, Copenhagen, Denmark

^2^Evolva SA, Duggingerstrasse 23, 4153 Reinach, Switzerland

^*^ Corresponding author^:^ Evolva SA, Duggingerstrasse 23, 4153 Reinach, Switzerland

# Supplementary materials

## Additional file 1: Figures


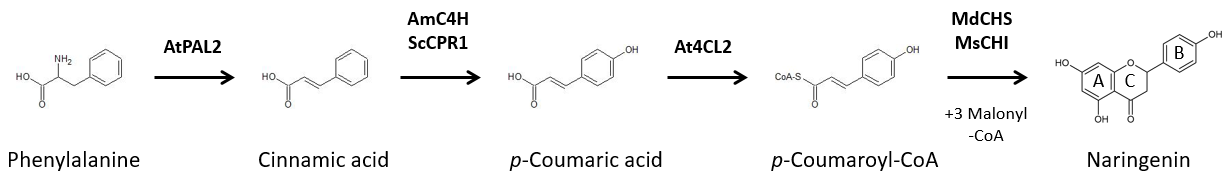


**Additional file 1: Figure S1:** *De novo* naringenin pathway in *S. cerevisiae* derived from phenylalanine and three malonyl-CoA [1].


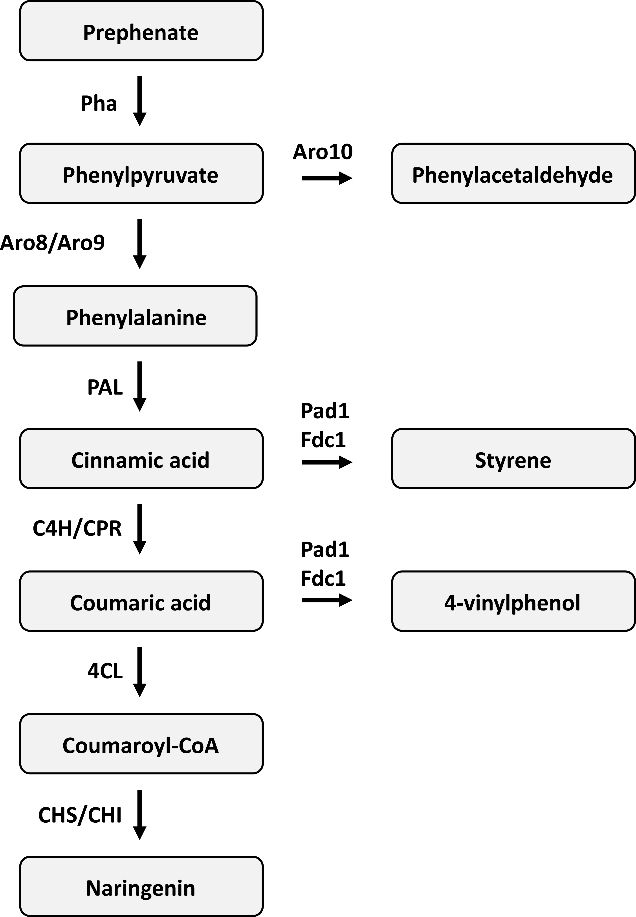


**Additional file 1: Figure S2:** Host modifications in the naringenin strain. *ARO10* encodes a phenylpyruvate decarboxylase which catalyses the conversion of phenylpyruvate to phenylacetaldehyde, the first specific step in the Ehrlich pathway. Phenylpyruvate is also the direct precursor of phenylalanine, which again is one of the two essential precursors for naringenin biosynthesis. The *ARO10* gene deletion prevents the loss of the essential phenylpyruvate precursor. Pad1 and Fdc1 are involved in the decarboxylation of aromatic carboxylic acids such as cinnamic and coumaric acid and formation of their corresponding vinyl derivatives. Deletion of the *PAD1* and *FDC1* genes prevents degradation of cinnamic and coumaric acids by decarboxylation, and thereby preserves the pool of these intermediates for the biosynthesis of naringenin. The *PAD1* and *FDC1* genes are non-essential in yeast and the disruption of the Ehrlich pathway by *ARO10* deletion does not affect the growth of yeast under laboratory and industrial settings. The normal function of the Ehrlich pathway in yeast is to allow amino acids to be used as an alternative nitrogen source. In the laboratory yeast is normally fed with a more readily exploitable nitrogen source, e.g. in the form of ammonium sulphate. The deletions were done by a simple construct: UPtag-DR-URA3-DR-DOWNtag, where UPtag and DOWNtag denotes DNA fragments of approx. 400 bp with homology to the yeast genome, upstream and downstream, respectively, of the region to be deleted. The tags directed the integration of *URA3* by homologous recombination at the targeted site. The DR denotes short approx. 25 bp direct repeats. These DRs were used to loop out the *URA3*, after deletion of the target genes, i.e. *ARO10*, *PAD1*, and *FDC1*, as described earlier [2,3]. *PAD1* and *FDC1* genes are located in close proximity on chromosome IV of yeast, and were thus deleted in a single disruption of the relevant locus.

**
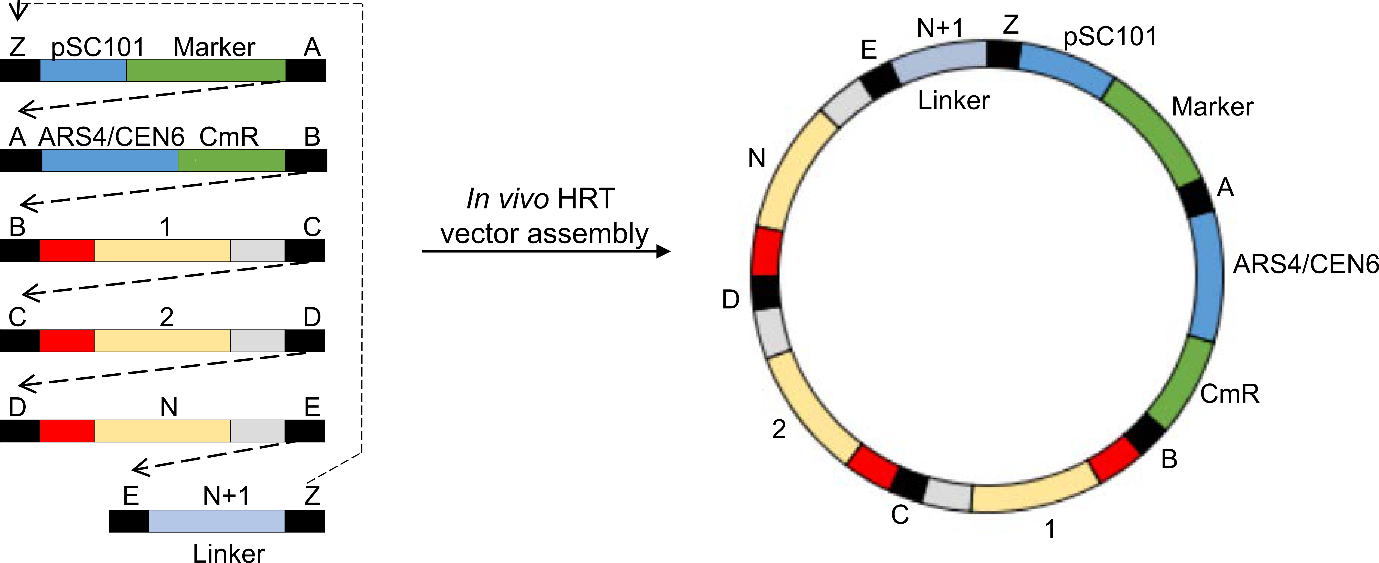
**

**Additional File 1: Figure S3:** One-step *in vivo* assembly using Homologous Recombination Technology (HRT) in *S. cerevisiae*. N represents the optional number of gene fragments. pSC101 is a bacterial origin of replication. Marker denotes the auxotrophic selection markers commonly used in yeast. The ARS4/CEN6 are the yeast signals for autonomous replication and segregation. CmR is a bacterial chloramphenicol selection gene for rescue of the plasmid for amplification in *E. coli*. B, C, D, etc. indicate 60 bps recombination tags for *in vivo* assembly by homologous recombination. The DNA fragments BC, CD, etc. comprise yeast expression cassettes with promoters (in red) and terminators (grey), into which the coding sequence of various enzymes (yellow) has been cloned. All DNA fragments are excised from a bacterial vector backbone (not shown) by *Asc*I restriction digest.

**Additional file 1: Figure S4:** Quantification of vitexin and isovitexin. A sample containing 1 mg/L of each reference compound was injected and EIC of mass 431.09839 was recorded. Left shoulder shows vitexin, right shoulder isovitexin. These results are in accordance with those previously reported by Nagatomo *et al.* 2014 [4]. Compounds were not baseline separated, but retention time in the instrument was very reproducible between samples, allowing quantification based on partial peak areas. A custom made script in MATLAB (MathWorks Inc., MA, USA) was used to quantify individual compounds, in this case using the left shoulder for vitexin and the right shoulder of the peak for isovitexin.

## Additional file 1: Tables

**Additional file 1: Table S1:** Synthetic and codon optimized genes used in this study.

| **Name** | **Enzyme class** | **Abbreviation** | **Source** | **Prot acc no.** |
| --- | --- | --- | --- | --- |
| *AtPAL2* | Phenylalanine ammonia-lyase | PAL | *Arabidopsis thaliana* | NP_190894.1 |
| *AmC4H* | Cinnamate 4-hydroxylase | C4H | *Ammi majus* | AA062904.1 |
| *ScCPR1* | Cytochrome P450 reductase | CPR | *Saccharomyces cerevisiae* | NP_011908.1 |
| *AtCPR1* | Cytochrome P450 reductase | CPR | *Arabidopsis thaliana* | AAC16897 |
| *At4CL2* | 4-Coumarate- CoA ligase | 4CL | *Arabidopsis thaliana* | NP_188761.1 |
| *HaCHS* | Chalcone synthase | CHS | *Hypericum androsaemum* | AAG30295.1 |
| *MsCHI* | Chalcone isomerase | CHI | *Medicago sativa* | AAB41524 |
| *AmCYP93B3* | Flavone synthase II (CYP450) | FNS II | *Antirrhinum majus* | BAA84071 |
| *OsCYP93G2* | Flavanone-2-hydroxylase | F2H | *Oryza sativa* | BAG94143 |
| *SbCYP93G3* | Flavanone-2-hydroxylase | F2H | *Sorghum bicolor* | XP_002461286 |
| *ZmCYP93G5* | Flavanone-2-hydroxylase | F2H | *Zea mays* | AFW88336 |
| *UGT708C2* | *C*-glycosyl transferase | CGT | *Fagopyrum esculentum* | BAP90361 |
| *UGT708D1* | *C*-glycosyl transferase | CGT | *Glycine max* | BAR73279 |
| *GtUF6CGT1* | *C*-glycosyl transferase | CGT | *Gentiana triflora* | BAQ19550 |
| *PhF3'H* | Flavonoid 3'-hydroxylase | F3'H | *Petunia x hybrida* | AAD56282.1 |

Coding sequences were based on the publicly available amino acid sequences. The corresponding protein accession numbers are provided.

**Additional file 1: Table S2:** Plasmids used in this study.

| **Plasmid**  **name** | **Content** | **HR tag** | **Backbone** | **Codon optimized** | **Source**  **organism** | **Description** |
| --- | --- | --- | --- | --- | --- | --- |
| pEVE4730 | Ura3_pSC101 | ZA | pUC18 | NA | NA | HRT plasmid |
| pEVE4729 | His3_pSC101 | ZA | pUC18 | NA | NA | HRT plasmid |
| pEVE1968 | ARS4/CEN6_CmR | AB | pUC18 | NA | NA | HRT plasmid |
| pEVE2176 | Stuffer I | BC | pUC18 | NA | NA | HRT backbone |
| pEVE2177 | Stuffer I | BC | pUC18 | NA | NA | HRT backbone |
| pEVE27453 | Stuffer II | CD | pEV2177 | NA | NA | HRT backbone |
| pEVE2178 | Stuffer I | DE | pUC18 | NA | NA | HRT backbone |
| pEVE1973 | Closing linker | CZ | pUC18 | NA | NA | HRT plasmid |
| pEVE1916 | Closing linker | EZ | pUC18 | NA | NA | HRT plasmid |
| pEVE4012 | *AtCPR1* | DE | pEVE2178 | yes | *Arabidopsis thaliana* | HRT plasmid |
| pEVE23312 | *AmFNSII* | BC | pEVE2176 | yes | *Anthirrhinum majus* | HRT plasmid |
| pEVE3999 | *PhF3'H* | CD | pEVE2177 | yes | *Petunia hybrida* | HRT plasmid |
| pEVE30360 | *OsCYP93G2* | CD | pEVE2177 | yes | *Oryza sativa* | HRT plasmid |
| pEVE30361 | *SbCYP93G3* | CD | pEVE2177 | yes | *Sorghum bicolor* | HRT plasmid |
| pEVE30362 | *ZmCYP93G5* | CD | pEVE2177 | yes | *Zea mays* | HRT plasmid |
| pEVE30366 | *FeUGT708C2* | BC | pEVE2176 | yes | *Fagopyrum esculentum* | HRT plasmid |
| pEVE30367 | *GmUGT708D1* | BC | pEVE2176 | yes | *Glycine max* | HRT plasmid |
| pEVE30369 | *GtUF6CGT1* | BC | pEVE2176 | yes | *Gentiana triflora* | HRT plasmid |

**Additional file 1: Table S3:** Indirect biosynthetic pathway.

| **Strains** | **Naringenin**  **[mg/l]** | **Eriodictyol**  **[mg/l]** | **Apigenin**  **[mg/l]** | **Luteolin**  **[mg/l]** | **Isovitexin**  **[mg/l]** | **Vitexin**  **[mg/l]** | **Isoorientin**  **[mg/l]** | **Orientin**  **[mg/l]** | ***p*-Coumaric acid**  **[mg/l]** | **Phloretic acid**  **[mg/l]** |
| --- | --- | --- | --- | --- | --- | --- | --- | --- | --- | --- |
| NCG1 | 2.21 ± 31.91 | -0.25 ± 0.04 | 4.93 ± 0.45 | -0.1 ± 0.03 | 140.33 ± 5.70 | 127.17 ± 4.96 | -0.53 ± 0.02 | -0.38 ± 0.02 | 23.92 ± 12.5 | 180.23 ± 14.04 |
| NCG2 | 1.73 ± 0.13 | -0.25 ± 0.05 | 16.53 ± 1.04 | -0.12 ± 0.02 | 91.65 ± 11.47 | 86.45 ± 10.38 | -0.55 ± 0.02 | -0.40 ± 0.02 | 37.20 ± 4.20 | 186.22 ± 9.48 |
| NCG3 | 2.23 ± 0.15 | -0.24 ± 0.03 | 5.28 ± 0.03 | -0.12 ± 0.02 | 142.87 ± 10.43 | 124.80 ± 10.13 | -0.53 ± 0.03 | -0.35 ± 0.01 | 22.29 ± 4.16 | 172.23 ± 3.18 |
| NCG4 | 1.51 ± 0.59 | -0.26 ± 0.02 | 20.45 ± 0.79 | -0.14 ± 0.00 | 71.15 ± 29.58 | 65.10 ± 25.33 | -0.57 ± 0.00 | -0.42 ± 0.04 | 35.38 ± 1.29 | 148.96 ± 37.50 |
| NCG5 | 2.34 ± 0.43 | -0.29 ± 0.03 | 5.82 ± 2.56 | -0.14 ± 0.01 | 119.20 ± 9.44 | 109.59 ± 6.23 | -0.56 ± 0.00 | -0.43 ± 0.03 | 22.49 ± 3.30 | 165.80 ± 5.81 |
| NCG6 | 1.76 ± 0.44 | -0.26 ± 0.03 | 16.21 ± 1.17 | -0.14 ± 0.01 | 97.00 ± 15.24 | 90.08 ± 10.5 | -0.56 ± 0.02 | -0.43 ± 0.01 | 37.18 ± 2.80 | 182.81 ± 18.52 |
| ECG1 | -0.2 ± 0.10 | 27.00 ± 0.90 | -0.19 ± 0.13 | 12.91 ± 6.67 | 51.38 ± 7.81 | 47.99 ± 5.96 | 26.81 ± 2.35 | 62.11 ± 5.79 | 30.31 ± 2.73 | 177.22 ± 13.41 |
| ECG2 | -0.24 ± 0.08 | 26.97 ± 2.62 | -0.31 ± 0.04 | 6.04 ± 0.01 | 49.77 ± 4.65 | 47.7 ± 3.40 | 29.19 ± 0.24 | 67.03 ± 2.92 | 23.47 ± 0.15 | 151.20 ± 3.78 |
| ECG3 | -0.21 ± 0.08 | 21.69 ± 5.9 | -0.23 ± 0.05 | 8.32 ± 1.04 | 71.84 ± 7.79 | 65.76 ± 8.87 | 21.51 ± 1.24 | 50.03 ± 3.05 | 27.58 ± 0.67 | 185.32 ± 4.43 |
| ECG4 | -0.27 ± 0.07 | 24.29 ± 0.85 | -0.28 ± 0.04 | 6.58 ± 0.37 | 70.21 ± 7.47 | 65.18 ± 5.54 | 25.03 ± 2.74 | 57.47 ± 7.02 | 20.80 ± 0.68 | 165.62 ± 14.77 |
| ECG5 | -0.26 ± 0.23 | 30.48 ± 2.00 | -0.27 ± 0.05 | 9.64 ± 0.98 | 58.12 ± 6.32 | 52.48 ± 4.29 | 27.90 ± 1.74 | 65.38 ± 5.16 | 31.40 ± 0.27 | 177.98 ± 10.08 |
| ECG6 | 0.27 ± 0.75 | 27.22 ± 5.00 | -0.29 ± 0.06 | 5.53 ± 1.38 | 56.17 ± 8.86 | 52.61 ± 6.72 | 24.11 ± 8.3 | 55.53 ± 19.03 | 30.01 ± 17.99 | 148.94 ± 26.28 |

Concentration of intermediates and products for the production of *C*-glycosylated flavones via the indirect pathway.

**Additional file 1: Table S4:** Additional intermediates of the indirect biosynthetic pathway.

| **Strains** | **2-hydroxynaringenin**  **[Area Under Curve]** | **2-hydroxyeriodictyol**  **[Area Under Curve]** | **2-hydroxynaringenin-*C*-glucoside**  **[Area Under Curve]** | **2-hydroxyeriodictyol-*C*-glucoside**  **[Area Under Curve]** |
| --- | --- | --- | --- | --- |
| NCG1 | 159.801 ± 36.008 | 8.404 ± 1.057 | 12.432.469 ± 1.303.757 | 19.411 ± 7.947 |
| NCG2 | 158.412 ± 40.202 | 8.174 ± 1.905 | 10.014.248 ± 535.108 | 15.644 ± 6.469 |
| NCG3 | 164.374 ± 26.021 | 8.395 ± 939 | 10.769.815 ± 1.135.835 | 19.385 ± 6.418 |
| NCG4 | 148.912 ± 16.268 | 7.363 ± 1.763 | 11.858.078 ± 6.300.457 | 16.675 ± 5.607 |
| NCG5 | 121.408 ± 29.049 | 8.596 ± 1.912 | 14.952.653 ± 1.096.428 | 18.597 ± 8.443 |
| NCG6 | 146.658 ± 27.613 | 8.853 ± 746 | 9.555.261 ± 526.430 | 17.713 ± 1.932 |
| ECG1 | 23.978.910 ± 785.328 | 17.850 ± 1.486 | 6.101.042 ± 612.326 | 12.801.996 ± 835.280 |
| ECG2 | 23.955.315 ± 2.291.739 | 16.470 ± 1.233 | 4.967.150 ± 133.528 | 13.130.645 ± 42.691 |
| ECG3 | 19.338.173 ± 5.150.865 | 50.120 ± 5.993 | 6.700.381 ± 1.523.018 | 10.272.848 ± 840.620 |
| ECG4 | 21.607.670 ± 743.787 | 56.023 ± 1.890 | 7.560.863 ± 1.087.072 | 12.464.939 ± 474.918 |
| ECG5 | 27.027.566 ± 1.472.809 | 11.226 ± 1.971 | 4.699.340 ± 970.446 | 12.324.263 ± 427.015 |
| ECG6 | 24.175.117 ± 4.378.493 | 12.245 ± 1.118 | 8.035.190 ± 4.376.949 | 13.004.223 ± 390.389 |

Semi quantitative results of putative 2-hydroxyflavanones and 2-hydroxyflavanones-*C*-glucoside intermediates.

**Additional file 1: Table S5:** Direct biosynthetic pathway.

| **Strains** | **Apigenin**  **[mg/l]** | **Isovitexin [mg/l]** | **Vitexin**  **[mg/l]** | **Luteolin**  **[mg/l]** | **Isoorientin [mg/l]** | **Orientin**  **[mg/l]** |
| --- | --- | --- | --- | --- | --- | --- |
| API2 (control) | 102.40 ± 9.84 | 0.00 ± 0.03 | 0.00 ± 0.03 | 0.03 ± 0.01 | -0.01 ± 0.04 | 0.00 ± 0.07 |
| ACG1 (with  GtUF6CGT1) | 65.75 ± 8.79 | 206.47 ± 9.70 | 4.99 ± 0.18 | 0.00 ± 0.02 | -0.03 ± 0.02 | 0.01 ± 0.03 |
| LUT2 (control) | 39.04 ± 1.74 | 0.09 ± 0.05 | 0.00 ± 0.01 | 62.94 ± 6.84 | 0.00 ± 0.02 | 0.00 ± 0.02 |
| LCG1 (with  GtUF6CGT1) | 5.86 ± 0.42 | 64.24 ± 0.51 | 1.63 ± 0.04 | 63.61 ± 4.28 | 31.47 ± 0.38 | 3.76 ± 0.13 |

Concentration of intermediates and products for the production of *C*-glycosylated flavones via the direct pathway.

## References:

1. Eichenberger M, Hansson A, Fischer D, Dürr L, Naesby M. De novo biosynthesis of anthocyanins in Saccharomyces cerevisiae. FEMS Yeast Res. [Internet]. 2018;1–13. Available from: https://academic.oup.com/femsyr/advance-article/doi/10.1093/femsyr/foy046/4975775

2. Baudin A, Ozier-kalogeropoulos O, Denouel A, Lacroute F, Cullin C. A simple and efficient method for direct gene deletion in Saccharomyces cerevisiae. Nucleic Acids Res. 1993;21:3329–30.

3. Wach A, Brachat A, Pöhlmann R, Philippsen P. New heterologous modules for classical or PCR‐based gene disruptions in Saccharomyces cerevisiae. Yeast. 1994;10:1793–808.

4. Nagatomo Y, Usui S, Ito T, Kato A, Shimosaka M, Taguchi G. Purification, molecular cloning and functional characterization of flavonoid C-glucosyltransferases from Fagopyrum esculentum M. (buckwheat) cotyledon. Plant J. 2014;80:437–49.
